# Allogeneic morphogenetic protein vs. recombinant human bone morphogenetic protein-2 in lumbar interbody fusion procedures: a radiographic and economic analysis

**DOI:** 10.1186/1749-799X-8-49

**Published:** 2013-12-28

**Authors:** Jeffrey S Roh, Christopher A Yeung, Justin S Field, R Trigg McClellan

**Affiliations:** 1 Orthopedics International, 12333 NE 130th Lane #400, Kirkland, WA 98034, USA; 2 Desert Institute for Spine Care, 1635 E Myrtle Ave, Phoenix, AZ 85020, USA; 3 Orthopaedic Trauma Institute, San Francisco General Hospital, UCSF, 2550 23rd Street, San Francisco, CA 94110, USA

**Keywords:** rhBMP-2, Spine, Bone graft, Allograft, Protein, Interbody, Fusion, OsteoAMP

## Abstract

**Background:**

Since the introduction of rhBMP-2 (Infuse®) in 2002, surgeons have had an alternative substitute to autograft and its related donor site morbidity. Recently, the prevalence of reported adverse events and complications related to the use of rhBMP-2 has raised many ethical and legal concerns for surgeons. Additionally, the cost and decreasing reimbursement landscape of rhBMP-2 use have required identification of a viable alternative. Osteo allogeneic morphogenetic protein (OsteoAMP®) is a commercially available allograft-derived growth factor rich in osteoinductive, angiogenic, and mitogenic proteins. This study compares the radiographic fusion outcomes between rhBMP-2 and OsteoAMP allogeneic morphogenetic protein in lumbar interbody fusion spine procedures.

**Methods:**

Three hundred twenty-one (321) patients from three centers underwent a transforaminal lumbar interbody fusion (TLIF) or lateral lumbar interbody fusion (LLIF) procedure and were assessed by an independent radiologist for fusion and radiographically evident complications. The independent radiologist was blinded to the intervention, product, and surgeon information. Two hundred and twenty-six (226) patients received OsteoAMP with autologous local bone, while ninety-five (95) patients received Infuse with autologous local bone. Patients underwent radiographs (x-ray and/or CT) at standard postoperative follow-up intervals of approximately 1, 3, 6, 12, and 18 months. Fusion was defined as radiographic evidence of bridging across endplates, or bridging from endplates to interspace disc plugs. Osteobiologic surgical supply costs were also analyzed to ascertain cost differences between OsteoAMP and rhBMP-2.

**Results:**

OsteoAMP produced higher rates of fusion at 6, 12, and 18 months (*p* ≤ 0.01). The time required for OsteoAMP to achieve fusion was approximately 40% less than rhBMP-2 with approximately 70% fewer complications. Osteobiologic supply costs were 80.5% lower for OsteoAMP patients (73.7% lower per level) than for rhBMP-2.

**Conclusions:**

Results of this study indicate that OsteoAMP is a viable alternative to rhBMP-2 both clinically and economically when used in TLIF and LLIF spine procedures.

## Background

Autograft, harvested from the iliac crest, has long been considered the ‘gold standard’ for spinal fusion. Though the use of autograft is well studied, limited tissue availability, donor site morbidity, and increased surgical time are also well understood [1]. In 1965, Dr. Marshal Urist discovered trace amounts of bone morphogenetic proteins (BMPs) naturally found within the bone matrix. Since this discovery, a wide range of allogeneic bone grafts has become available as a substitute or extender to autograft [2], yet with limited success. The goal of these allogeneic bone grafts was to offer the greatest amount of BMP available within the tissue, but these bone graft products are constrained by the small amount of BMPs found within the actual collagen matrix [3].

In 2002, the FDA approved the use of a recombinant human bone morphogenetic protein-2 (Infuse, Medtronic Inc., Fridley, MN, USA) for single-level anterior lumbar interbody fusion (ALIF) spine surgery. The initial success of rhBMP-2 with interbody fusion soon led to off-label use, beyond the initial FDA indication [4]. Unfortunately, many adverse events have been reported stemming from its use, including retrograde ejaculation, dysphagia, ectopic bone formation and potentially cancer, leading clinicians to question the clinical benefit versus patient safety [4-7]. In 2013, the Yale University Open Data Access (YODA) Project determined that there was no increased incidence of retrograde ejaculation, though the potential increase in cancer was higher in Infuse patients. The YODA study indicated that the increased cancer incidence with rhBMP-2 was 1.9%-3% [8].

The discovery of a new allogeneic tissue processing technique has provided a way to access BMPs and other growth factors that are naturally found within bone marrow cells. OsteoAMP (Advanced Biologics Carlsbad, CA, USA) is an allogeneic morphogenetic protein that undergoes a novel tissue processing technique, utilizing angiogenic, mitogenic and osteoinductive growth factors such as BMP-2, BMP-7, TGF-β1, aFGF, VEGF, and ANG1, within bone marrow cells [9-11] and naturally binds them back to the bone graft being processed. This array of growth factors, beneficial to bone growth, could offer an alternative to rhBMP-2 and other current bone graft substitutes on the market today.

The objective of this study was to compare OsteoAMP allogeneic morphogenetic protein to rhBMP-2 by examining the radiographic evidence of spinal fusion at various time points in patients who have undergone lumbar interbody fusion and to also examine the costs associated with the use of these products.

## Methods

A dual-arm radiographic analysis was conducted at three clinical sites to evaluate the fusion success rate of a commercially available allogeneic morphogenetic protein (OsteoAMP) and rhBMP-2 (Infuse) in lumbar spine surgery. Radiologic review and evaluation were conducted by a blinded independent radiologist for interbody fusion. The indications for surgery were symptomatic patients diagnosed with degenerative disc disease (DDD), stenosis, and/or spondylolisthesis (Table 1). Surgical supply costs for the osteobiologics used were also analyzed to present an overall value analysis between the two arms.

**Table 1:** Pathology breakdown

**Pathology breakdown**	**OsteoAMP (%) (*n*** **= 226)**	**rhBMP-2 (%) (*n*** **= 95)**
DDD	21.7	26.3
Herniated disc	25.7	2.1
Pseudarthrosis/nonunion/hardware failure/revision	5.3	6.3
Post laminectomy/fusion syndrome	21.2	1.1
Radiculitis/radiculopathy	51.3	1.1
Scoliosis	11.1	10.5
Spondylosis	8.8	0.0
Spondylolisthesis	74.8	75.8
Stenosis	58.0	17.9

Patients presented with multiple pathologies; thus, the percentage will not equal 100.

### Patient demographics

Three hundred twenty-one consecutive patients over the course of 5 years underwent transforaminal lumbar interbody fusion (TLIF) or lateral lumbar interbody fusion (LLIF) between T4 and S1 (522 operative levels/501 interbody fusion levels). A group of 95 patients with a mean age of 54.3 were treated with rhBMP-2 with an average of 1.63 levels per surgery. A group of 226 patients with a mean age of 60.0 were treated with OsteoAMP with an average of 1.62 levels per surgery (Table 2). On average, patients in the OsteoAMP arm were 10.5% older than the patients in the rhBMP-2 cohort.

**Table 2:** Patient baseline characteristics

**Characteristic**	**OsteoAMP**	**rhBMP-2**
	**(*n*** **= 226)**	**(*n*** **= 95)**
Age, mean ± SD	60.0 ± 13.0	54.3 ± 10.9
Female, *n* (%)	131 (58.0)	56 (58.9)
Average levels/case	1.62	1.63
Operative levels, *n* (%)		
One	147 (65.0)	72 (75.8)
Two	58 (25.7)	14 (14.7)
Three	9 (4.0)	1 (1.1)
Four	4 (1.8)	2 (2.1)
Five	3 (1.3)	1 (1.1)
Six	0 (0.0)	0 (0.0)
Seven	3 (1.3)	3 (3.2)
Eight	1 (0.4)	1 (1.1)
Twelve	1 (0.4)	1 (1.1)

### Materials

Both arms utilized morselized local bone from the surgical site in combination with the osteobiologic. In the first arm, OsteoAMP was used in conjunction with the centers' preferred spinal spacer and fixation system. OsteoAMP was prepared for use per the instructions by the manufacturer (Advanced Biologics). In 58.4% of the OsteoAMP with local bone cases, bone marrow aspirate (BMA) was also added. In the second arm, rhBMP-2 was obtained and loaded onto the absorbable collagen sponge (ACS) as directed by the instructions for use. The rhBMP-2 was then combined with local autologous bone and packed into an interbody device. On average, approximately 3.07 mg of rhBMP-2 was used inside the interbody device per level.

### Analysis

Patients underwent radiographs (x-ray and/or CT) at standard postoperative follow-up time points, which were generally at 6, 13, 26, 52, and 72 weeks postsurgical procedure. In both arms, the majority of fusion assessments at each time point were made using static radiographs (Table 3). Although CT is widely accepted as the standard for noninvasive assessment of spinal fusion, the increased radiation exposure and limited equipment availability make it impractical to subject patients to CT at each visit [12]. When evaluated appropriately, literature has shown x-rays to have similar accuracy when assessing fusion [13].

**Table 3:** Radiologic breakdown for fusion

**Radiologic breakdown**	**OsteoAMP (*n*** **= 226)**	**rhBMP-2 (*n*** **= 95)**
Static x-ray	161	38
Computer tomography	65	57

**Figure 1:**
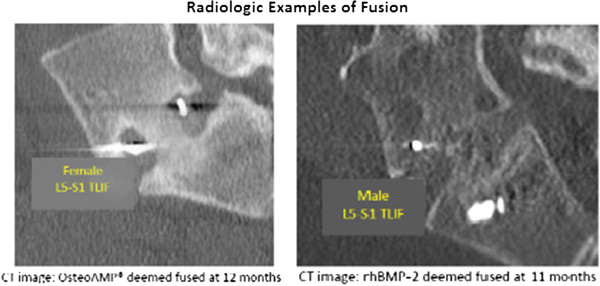
Radiologic examples of fusion.

An independent radiologist made fusion assessments based on these studies and was blinded to intervention, product, and surgeon information. Fusion was defined as any radiographic evidence of bridging across endplates, or bridging from endplates to interspace disc plugs (Figure 1). Any radiodensity that obliterates or blurs the lucency between endplates and plugs that was seen on the postoperative films was considered an evidence of fusion. Fusion success rate of each patient was then analyzed for both study arms at each time point. The time frame between surgical intervention and positive fusion assessment was calculated and radiologically evident complications of osteolysis or ectopic bone formation were reported. Osteobiologic surgical supply costs were determined by reporting cost of the implant utilized inside the spinal spacer or interbody device. If BMA was utilized, the cost of the jamshidi needle was included in the overall cost. In addition, a secondary analysis was conducted within the first arm to identify any correlation between the use of BMA and fusion success.

## Results

### Fusion success rate

**Figure 2:**
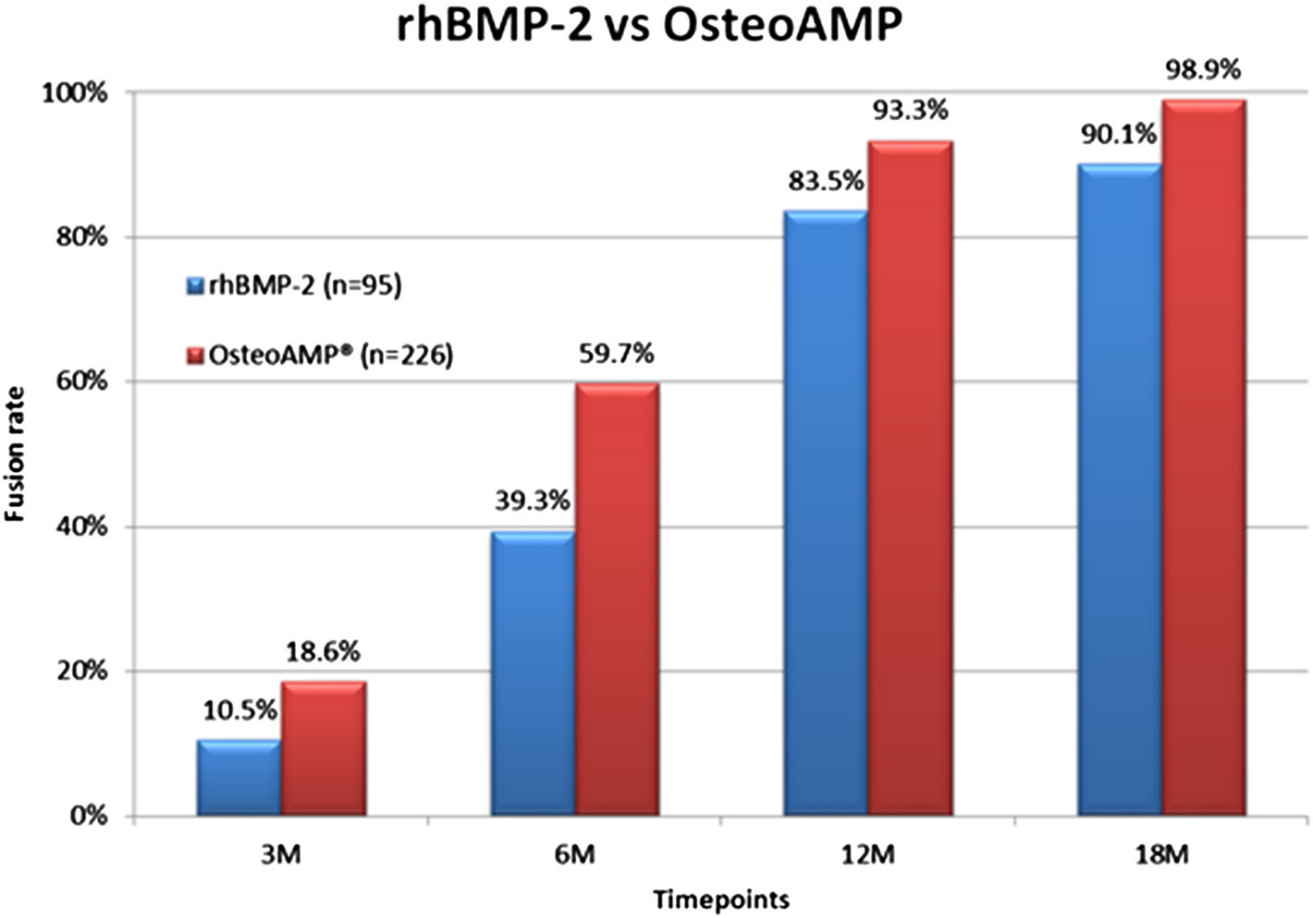
TLIF/LLIF fusion rates.

The series of radiographs from each patient were evaluated comparing postoperative x-rays and each consecutive time point for evidence of fusion to ensure that the opacity of the biologic or local bone was not a factor in the fusion assessment. The comparison of both arms demonstrated a statistically significant difference in the increase of fusion over each consecutive follow-up time point. Of the 226 patients receiving OsteoAMP, the radiographic fusion analysis indicated that there was fusion in 59.7% at 6 months, 93.3% at 12 months, and 98.9% at 18 months. Of the 95 patients receiving rhBMP-2, the analysis indicated that there was fusion in 39.3% at 6 months, 83.5% at 12 months, and 90.1% at 18 months (Table 4). The results indicated a higher percentage of patients fused in the OsteoAMP group at all time points (*p* ≤ 0.01) (Figure 2). Total time for fusion for OsteoAMP was approximately 40% shorter than that of rhBMP-2 (207.9 and 333.9 days, respectively).

**Table 4:** Fusion success at each time point

**Time point fusion (months)**	**OsteoAMP (%) (*n =*** **226)**	**rhBMP-2 (%) (*n =*** **95)**
3	18.6	10.5
6	59.7	39.3
12	93.3	83.5
18	98.9	90.1

### BMA analysis

**Figure 3:**
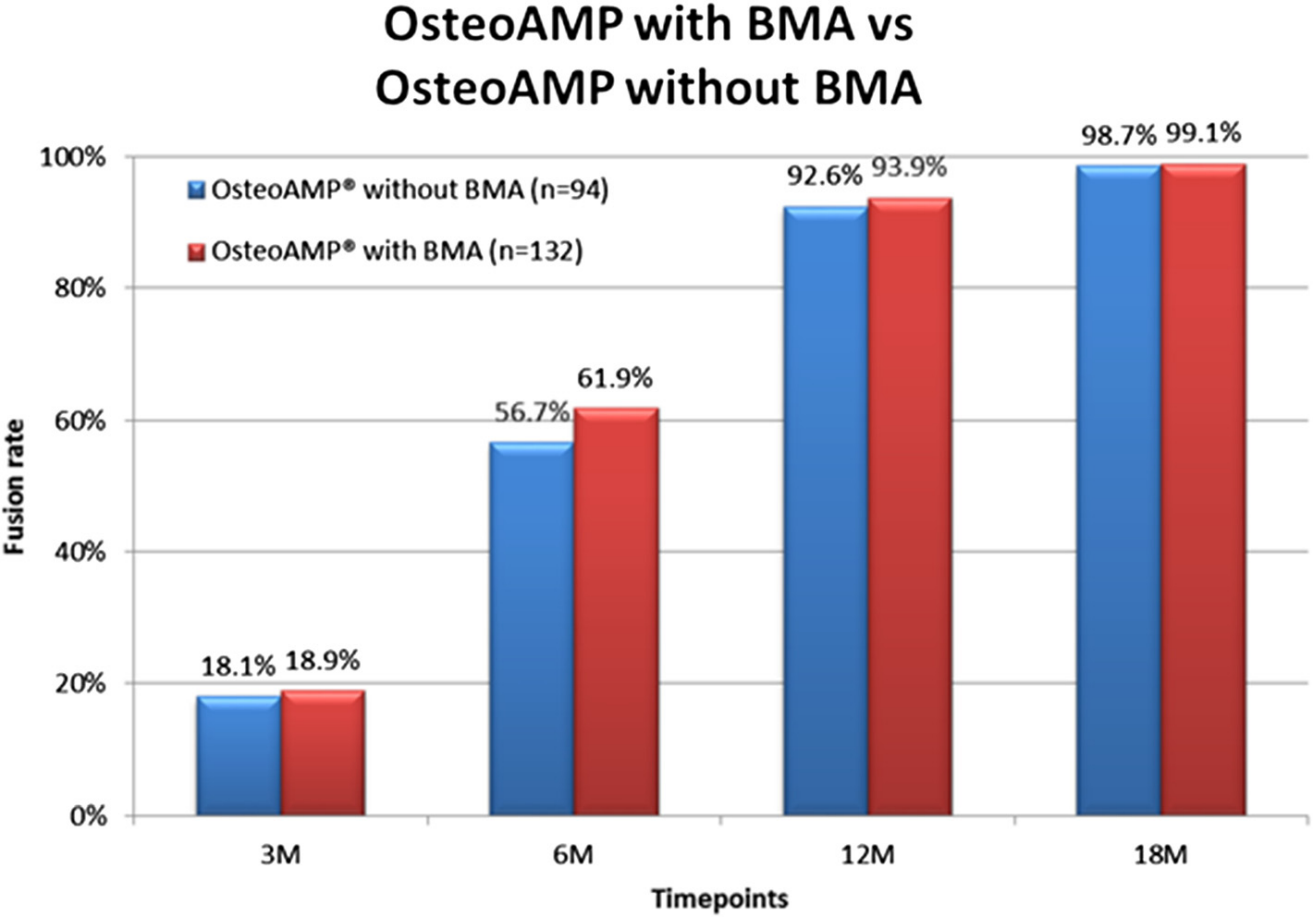
OsteoAMP with and without BMA.

Of the 132 patients receiving OsteoAMP with BMA, the radiographic fusion analysis determined that 61.9% of patients were fused at 6 months, 93.9% at 12 months, and 99.1% at 18 months. Of the 94 patients receiving OsteoAMP without BMA, the analysis indicated that 56.7% fused at 6 months, 92.6% at 12 months and 98.7% at 18 months (Table 5). The difference between groups was not statistically significant (*p* = 0.44 at 6 months, *p* > 0.72 at 12 and 18 months) (Figure 3).

**Table 5:** Fusion success at each time point (BMA)

**Time point (months)**	**OsteoAMP® with BMA (%) (*n*** **= 132)**	**OsteoAMP® without BMA (%) (*n*** **= 94)**
3	18.9	18.1
6	61.9	56.7
12	94.7	92.6
18	99.1	98.7

### Osteobiologic surgical supply cost analysis

The 95 patient rhBMP-2 arm utilized an average of 2.05 cc (3.07 mg rhBMP-2) inside the interbody device per level. The supply costs associated with rhBMP-2 was US$2,523.52 per level. The OsteoAMP arm utilized an average of 2.5 cc per level inside the spinal spacer. The supply costs associated with OsteoAMP was US$649.20 per level. The addition of a jamshidi needle used for BMA collection brought the OsteoAMP weighted cost per level to US$663.61. The OsteoAMP arm was 80.5% less expensive per patient (73.7% per level) than the rhBMP-2 arm.

### Complications

**Figure 4:**
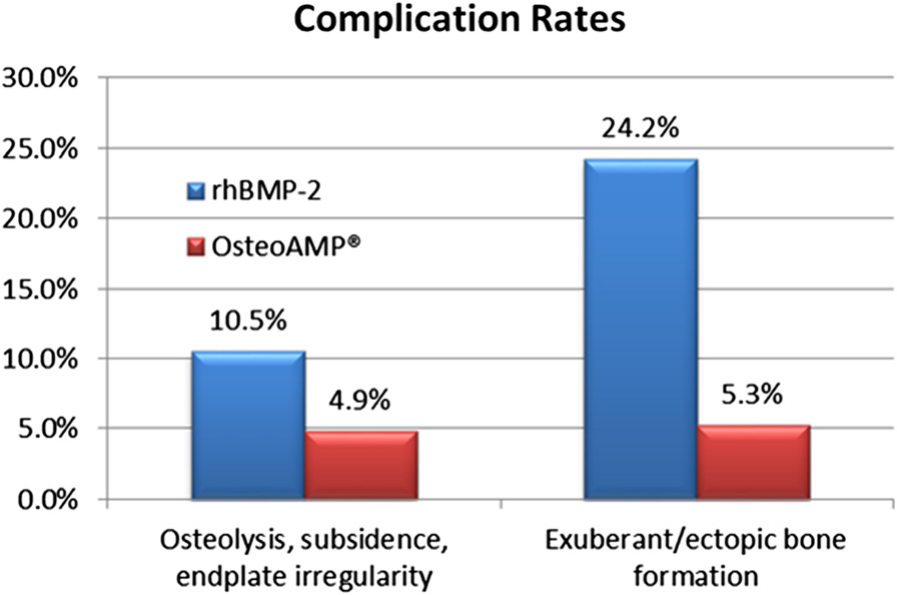
Complication rates.

The observation of complications was noted by reviewing x-rays, CT reports, general radiological assessment, and surgeon's notes. The rhBMP-2 arm seemed to have greater radiologically evident adverse events compared to the OsteoAMP arm. Radiologic evidence of ectopic bone formation was found in 24.2% of the rhBMP-2 cases compared to 5.3% in patients receiving OsteoAMP (*p* < 0.01). There was also greater radiologic evidence of osteolysis, subsidence, or endplate irregularity in the patients receiving rhBMP-2 (10.5%) versus OsteoAMP (5.3%) (*p* = 0.06) (Figure 4). It should be noted that the complication observations in both study arms could be underreported due to the use of other methods besides CT (standard method) to assess complications.

## Discussion

Long-term success following lumbar fusion procedures is predicated on the integrity of the fused interbody segments, yet the literature reports that nonunions occur in 10% to 20% of patients with single-level fusions [14-17]. Autograft from the iliac crest has been used successfully; however, morbidity, limited supply, and additional operating time associated with autograft harvest have led surgeons to consider alternatives.

The evolution of bone graft materials, advanced implant designs, and less invasive surgical techniques has greatly improved clinical outcomes [18-20]. There are many bone graft alternatives today, each with advantages and disadvantages. These bone graft alternatives will become more highly refined, as our knowledge continues to improve regarding the biology and mechanics of spinal fusion [14,21].

The introduction of rhBMP-2 in 2002 provided access to large quantities of BMP that is only available in trace amounts in demineralized bone or autograft [22]. Successful fusion results of rhBMP-2 when used in anterior lumbar interbody fusions are well documented [23,24]. As surgeons began to incorporate more minimally invasive techniques, the off-label use of rhBMP-2 has increased. In addition, the use in cervical procedures has led to serious complications [25]. Since then, there have been many attempts to facilitate bone healing and new bone growth to the same degree as with rhBMP-2. More recently, the use of stem cells, harvested from cadavers and frozen prior to surgery, has attempted to deliver viable cells directly to the surgical site [26]. While there are some reports of successful outcomes in the literature [27], the question still remains regarding the efficacy of such products [28].

In 2009, OsteoAMP was made commercially available. Utilizing a novel tissue processing technique, the naturally occurring growth factors and BMPs from bone marrow cells are harvested and naturally bound to the cadaver bone providing a product with angiogenic, mitogenic, and osteoinductive properties.

In a retrospective study from three centers over a 5-year period, radiographic fusion (x-ray and CT) was compared between OsteoAMP and rhBMP-2. The rate of fusion assessed by an independent radiologist was higher in the OsteoAMP group at all intervals (*p* ≤ 0.01). Although adverse events were only determined by radiological review in this study, OsteoAMP had dramatically fewer complications than rhBMP-2. These results seem to support the benefits of having an array of growth factors that are found in OsteoAMP. Bone remodeling involves factors, such as insulin-like growth factors (IGFs), BMPs, and vascular endothelial growth factor (VEGF) to not only stimulate cell recruitment and proliferation but also encourage vascularization [29,30]. OsteoAMP is processed so that these growth factors are made bioavailable.

Since standard practice at all three centers included the use of BMA, data was analyzed to identify the efficacy of BMA. When comparing the fusion results within the OsteoAMP patient population with and without BMA, data suggests little difference in fusion success with or without BMA.

Preparation of OsteoAMP is similar to other bone substitutes. The product is often reconstituted with blood or BMA to help with the handling characteristics prior to use. Unlike rhBMP-2, there is no time required for growth factor binding. However, aspiration of bone marrow will be dependent on surgical technique and could be difficult to mix with the graft if allowed to congeal in the aspiration syringe.

The inherent risks with the use of allograft tissue and bovine collagen have been discussed in literature for many years. Allogeneic materials are widely used in orthopedics; according to the Centers for Disease Control and Prevention, there have been no reports of disease transmission during the 30-year history of using freeze-dried bone allografts. However, there have been reports of disease transmission in nonsterile fresh-frozen bone allografts [31], and literature reports that 3% to 5% of the population is hypersensitive to bovine collagen [32,33]. The established exclusionary criteria combined with recommended processing procedures established by the American Academy of Tissue Banking have ensured that freeze-dried bone allografts are safe for human implantation [34,35].

There were a number of potential limitations in this study. Each center used different instrumentation and fixation devices, which may influence some of the results. The study did not evaluate the clinical outcomes, and follow-up CTs as well as x-rays were used to assess fusion over each time point. In addition, the three surgeons have unique surgical techniques that may have contributed to some variability within the results.

## Conclusions

The results of this study indicated a higher percentage of patients fused in the OsteoAMP group at all time points (*p* ≤ 0.01) (Figure 2). Furthermore, the OsteoAMP arm was shown to be 80.5% less expensive per patient (73.7% per level) than the rhBMP-2 arm. The economic analysis and superior fusion rate results in this study support OsteoAMP as a cost-effective and viable alternative to rhBMP-2. Multicenter randomized controlled studies will be necessary to confirm the efficacy and cost-effectiveness of OsteoAMP as a superior osteobiologic.

## Consent

When necessary, written informed consent was obtained from the patient for the publication of this report and any accompanying images.

## Competing interests

TM is an unpaid consultant for Advanced Biologics. Authors JR, CY, and JF are unpaid consultants for Advanced Biologics and hold shares in the company.
